# Trust in adolescence: Development, mechanisms and future directions

**DOI:** 10.1016/j.dcn.2024.101426

**Published:** 2024-08-02

**Authors:** Lydia Krabbendam, Hester Sijtsma, Eveline A. Crone, Mariët van Buuren

**Affiliations:** a Department of Clinical, Neuro, and Developmental Psychology, Institute for Brain and Behavior Amsterdam, Vrije Universiteit Amsterdam, the Netherlands; b Erasmus School of Social and Behavioral Sciences, Erasmus University Rotterdam, the Netherlands

**Keywords:** Trust, Social reinforcement learning, Adolescence

## Abstract

Trust is the glue of society. While the trust we place in close others is crucial for our wellbeing, trust in strangers is important to fulfill needs that families and friends cannot provide. Adolescence is an important phase for the development of trust in strangers, because the social world of adolescents expands tremendously. We provide an overview of the development of trust in adolescence by reviewing studies that used the trust game, an experimental paradigm to measure trust between dyads during monetary exchange. We start from the notion that trust is a form of social reinforcement learning in which prior beliefs about the trustworthiness of others are continuously updated by new information. Within this framework, development in adolescence is characterized by increasing uncertainty of prior beliefs, a greater tolerance of uncertainty, and a greater tendency to seek and use new information. Accordingly, there is evidence for an increase in initial trust and better adaptation of trust during repeated interactions. Childhood psychological and social-economic adversity may impact this development negatively. To further our understanding of these individual differences, we suggest ways in which the trust game can be enriched to capture trust dilemmas that are relevant to youth with diverse backgrounds.

## 1 Background

Trust is fundamental to successful social interactions. Trusting others and being trusted are associated with important benefits, both for the individual and for society as a whole. But trust also involves a risk: it may be costly to trust someone who is untrustworthy. It is therefore crucial to be able to judge the trustworthiness of others. Trust in close others, also known as ingroup trust or thick trust, is intuitive and ubiquitous (Putnam, 2000). Based on affective bonds, it facilitates fundamental needs of identity and belonging. However, in modern societies, we cannot rely solely on trust in people who are close to us. Trusting people we do not know, also called outgroup trust or thin trust, has become an essential part of our daily lives, in economic, cultural, and institutional domains (Misztal, 1996).

How do we learn to trust strangers? Evidence from the World Values Survey across fifty societies suggests that self-reported outgroup trust develops from self-reported ingroup trust, but is also partially independent (Delhey and Welzel, 2012). In other words, ingroup trust is a necessary, but not a sufficient condition for outgroup trust. Outgroup trust will only develop when the environment offers sufficient opportunity for cooperative experiences with outgroup members (Delhey and Welzel, 2012). This freedom to form connections with other people regardless of their background characterizes modern societies, and contributes to human empowerment in the economic, cultural and institutional domain (Delhey and Welzel, 2012). Accordingly, outgroup trust is lower in countries with high-income inequality (Jordahl, 2007) and in individuals with lower educational qualifications (OECD., 2015). Outgroup trust is strategic, rather than intuitive, and may require deliberate processes to be initiated and maintained (Krueger and Meyer-Lindenberg, 2019).

Early trust interactions during childhood are thought to serve as a foundation for later trust interactions with peers and strangers (Stolle, 2002). Erikson's model (Erikson, 1963) on psychosocial development already described the importance of the first stage of life (called ‘Trust vs. mistrust’ during the first 18 months after birth) in which infants are fully dependent on their caregivers for basic needs and care, laying the foundation for the development of trust. Relatedly, the attachment theory posits that trust emerges out of a secure parent-child relationship, and these parent-child trust experiences may translate and extend to relationships later in life (Bowlby, 1969). Building on these theories, there is now a rich literature documenting the importance of early relationships for later social-emotional outcomes Delgado et al., 2022, Feeney et al., 2008, Kochanska et al., 2019, Sutton, 2019. During adolescence, a reorientation towards peers and engagement in more diverse social networks takes place, which provides both the opportunity and the need to trust strangers. This social reorientation is facilitated by the ongoing maturation of (social) cognitive skills, the increased sensitivity to socially salient stimuli, and the associated brain development (Nelson et al., 2016), making adolescence arguably the key phase for the development of trust outside the circle of caregivers and families. Adolescence is also the period when the onset of psychopathology peaks (Guyer, 2020). Many forms of psychopathology have in common a marked impairment in the development of successful social relationships. Understanding the development of trust in adolescence is therefore also important from a clinical perspective.

In what follows, we first present our general framework of trust and development of trust. We then review how the ability to trust and to adapt trust in the face of new information develops during adolescence, focusing on studies that have used the trust game (see Box 1). Based on the extant behavioral and neuroimaging studies, we discuss the behavioral and neural mechanisms that may underlie the development of initial trust and trust adaptation. In the final section, we reflect on the strengths and limitations of the trust game, and discuss several ways in which the trust game can be enriched to capture meaningful aspects of trust for youth with diverse backgrounds.

Box 1 **Measuring trust.**

**Table:** 

**Fig. 1:**
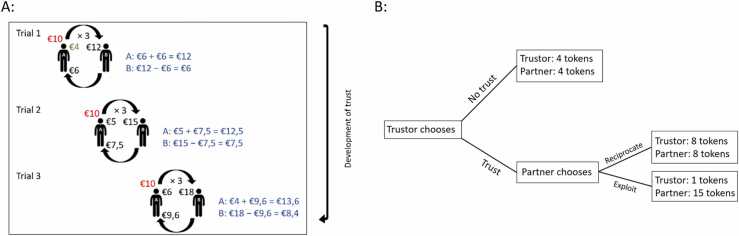
*The procedure of the trust game.* A: An example of a multi-round trust game with a trustworthy partner. In trial 1, A (the trustor) starts off with 10 euros (in red) of which 6 euros are kept and 4 euros are invested. Often, a multiplier of three is used which means that B (the partner) receives 12 euros (4 × 3). To control and simulate trustworthy or untrustworthy partner behavior, a preprogrammed algorithm can be applied. In this example, trustworthy partner behavior is simulated. The specific partner return in a trial can be determined by multiplying the investment by a return factor. In the first trial of this example, a return factor of 1.5 is used because the aim is to simulate trustworthy partner behavior. In this example, the partner returns 6 euros (4 × 1.5) in trial 1 which makes the outcome for this trial (in blue) 12 euros (6 euros + 6 euros) for A and 6 euros (12 euros – 6 euros) for B. In trial 2, A can decide to increase the investment (compared to trial 1) in response to B’s trustworthy behavior during trial 1 and thus to invest 5 euros, which is again tripled. B receives 15 euros of which 7,5 is shared with A (again, a return factor of 1.5 is used: 5 × 1.5). The outcome for trial 2 is 12,5 euros (5 euros + 7,5 euros) for A and 7,5 euros (15 euros – 7,5 euros) for B. The aim of the trust game is to simulate an interactive exchange so the algorithm can be manipulated to make the partner responsive to A’s behavior. A increased the investment in trial 2 with respect to trial 1 and therefore B can respond to this by showing increasingly trustworthy behavior in trial 3. In this example, this means that B’s return factor increases by 0.1 so the return factor in trial 3 is 1.6. In trial 3, A keeps 4 euros and invests 6 euros, which is tripled and thus B receives 18 euros. B returns 9,6 euros to A (a return factor of 1.6 is used: 6 × 1.6). The outcome for trial 3 is 13,6 euros (4 euros + 9,6 euros) for A and 8,4 euros (18 euros – 9,6 euros) for B. In a one-shot trust game (existing of one trial), the investment signals the level of initial trust behavior. In a multi-round trust game, the level of initial trust behavior can be measured by the investment in trial 1, indicated in green. By introducing multiple trials, as done in a multi-round trust game, a back and forth interaction arises in which the trustor and the partner respond to each other’s behavior and the development of trust appears (black arrow). Different elements of the game can be adapted, such as the return factor used to model trustworthy or untrustworthy partner behavior or the multiplier that is used when the investment is shared with the partner, leading to multiple variants of the trust game. B: An example of a binary choice trust game. In a binary choice trust game, the trustor is presented with a decision tree which includes a no-trust and a trust choice. In this example, the no-trust choice is an equal distribution of the tokens. The trust choice equals the trustor letting the partner decide how the tokens are distributed. In this case, the amount of tokens will be doubled and the partner can either reciprocate trust (i.e., by choosing an equal distribution of tokens) or exploit (i.e., by keeping more tokens oneself). Again, one or multiple trials can be used to study initial trust behavior and the development of trust over trials. Different elements of the binary choice trust game can be adapted as well, e.g., the amount of tokens that represent the no-trust and trust choice or whether the partner’s reciprocate and exploit options are visible for the trustor, leading to multiple variants of the game. The example in Fig. 1B is based on Van de Groep et al. (2018).

Large-scale population studies such as the World Value Survey (WVS) measure self-reported attitudes towards trust using non-incentivized questions. The WVS includes a binary question (“Generally speaking, would you say that most people can be trusted or that you need to be very careful in dealing with people?” with the answers options “Most people can be trusted” and “Need to be very careful.”) and a question on a 4-point scale. This latter question distinguishes ingroup and outgroup trust by asking whether people trust their family, friends and people they know personally versus people they meet for the first time, people of another religion, and people of another nationality. These longitudinal surveys have identified important trends, showing for example that income inequality and lower educational attainment are associated with lower self-reported trust, both across and within countries (Integrated Values Surveys, 2022). These surveys generally do not include children and early adolescents; however, age trends from late adolescence to late adulthood point to an increase of self-reported trust with age (Poulin and Haase, 2015). Zooming in on development during adolescence, one study of 1535 adolescents showed that middle and late adolescents had significantly lower levels of generalized trust than early adolescents (Flanagan and Stout, 2010 [ages 11–18]). This may suggest a U-shaped development of self-reported trust with an initial decline during adolescence. The strengths of the self-report approach include the very large samples across many countries and the longitudinal assessments. This allows for the identification of trends in trust beliefs over time and across societies. However, trust beliefs seem to be weak predictors of actual trust behaviors, but instead may reflect one’s own trustworthy behavior (Glaeser et al., 2000) and this ‘social projection’ seems to emerge during adolescence (De Neys et al., 2015 [ages 13–18]). In addition, self-report questionnaires cannot shed light on the dynamics of the development of trust in social interactions. An alternative approach is using experimental methods to measure trust. The trust game (Berg et al., 1995) is arguably the most widely used paradigm to elicit trust in an incentivized way. In this game, one player (the trustor) receives an amount of money and shares part of it with a second player (the trustee or the partner) (see Fig. 1A). The amount shared is called the investment and multiplied by a predefined factor (often three). After receiving the money from the trustor, the partner decides what part of this amount to return to the trustor. Investing can be beneficial to both, but is risky for the trustor, because of the possibility of a low return by the partner. The trustor’s investment is seen as a measure of trust and the partner’s return as a measure of reciprocity behavior. To ensure the game is comprehensible for children, developmental studies have also used binary trust choices with tokens instead of monetary incentives (see Fig. 1B) (Van de Groep et al., 2018). Some studies used the strategy vector method, instead of direct responses, which asks trustors to make decisions for different combinations of outcomes (Harbaugh et al., 2003). The strengths of the trust game, and other social dilemmas, include the strict experimental control, the suitability for use in functional imaging environments, and the opportunity to manipulate relevant task features to study their effect on trust behavior. Using this approach, researchers have been able to disentangle different processes underlying initial trust and the adaptation of trust, compare different targets of trust, study the neural mechanisms, and relate trust behavior to characteristics of the individuals and their environment.

## 2 The general framework of trust and development of trust

We start from the definition of trust as the willingness to be vulnerable to the behavior of another party, expecting a positive outcome in the future (Mayer et al., 1995). This definition captures a key element of trust, namely that the decision to trust is faced with uncertainty, since the response of the other cannot be predicted nor controlled. The decision is not made in a vacuum, however. It is shaped by many factors, including previous experiences of trust and distrust (Simpson, 2007), the judgment of the situation and of the other party (Hancock et al., 2023), attitudes toward risk (Fairley et al., 2016), and preferences regarding outcomes for self and other (Fehr et al., 2005). Together, these factors form the prior belief regarding the expected value of the action to trust another. The decision to trust is then evaluated: the actual outcome is compared to the expected outcome. If trust is reciprocated, the decision to trust is reinforced. If not, the decision to trust is discouraged. In this sense, learning to trust is similar to basic reinforcement learning, in which an agent learns the value of specific actions by comparing the expected outcome with the actual outcome. If there are discrepancies between the expected outcome and the actual outcome, a prediction error signal is generated which is used to update the expectations (Joiner et al., 2017). A better-than-expected outcome will reinforce the specific action, a worse-than-expected outcome will discourage the action.

In the case of trust, and many other forms of social learning, the expected outcome is uncertain. This uncertainty can be captured by Bayesian learning models, in which the expected value of a certain action is expressed as a probability distribution over a range of possible outcomes (Hofmans and Van den Bos, 2022). The degree of uncertainty influences how the prior belief is weighted against new information. If uncertainty is high, the prior expectation is relatively weak. As a consequence, new information will be assigned more weight, resulting in an update of the probability distribution. In contrast, if the prior expectation is precise, new information will have low impact on the resulting distribution of the expectation. In this paper, we show how this framework can contribute to our understanding of the development of trust in adolescence, including the differences in trust in adolescents from diverse social-economic backgrounds. Given the important benefits of trust for the individual and for society, understanding the barriers and facilitators of the development of trusting relationships is crucial.

## 3 The development of initial trust in adolescence

### 3.1 Behavioral studies

Initial trust is important. It is a first indication of the level of trust that is placed in (known or unknown) others and signals to the other the motivation to engage in a mutually beneficial interaction. It also shows the willingness to be vulnerable to the actions of the other, and placing high levels of initial trust can end up being harmful. Regardless of the outcome, initial trust provides the opportunity to learn about the trustworthiness of others. Low initial trust reduces the chance to build a trusting relationship and to learn about the other. Within the trust game, initial trust is measured as the investment in one-shot games, or sometimes conveniently as the first investment in multi-round games (see Fig. 1).

In their seminal study of trust and reciprocity in different age groups, Sutter and Kocher (2007) showed that initial trust behavior in anonymous one-shot trust games increases almost linearly from childhood (age 8) till early adulthood (age 22). This finding has been replicated in diverse samples, both in one-shot trust games (Van den Bos et al., 2010 [four age groups of 9, 12, 16, and 22 years]) and using the first investment in multi-round games (Reiter et al., 2023 [ages 14–25]; Sijtsma et al., 2023 [ages 12–16]; Van den Bos et al., 2012 [three age groups of 11, 16, and 21 years old]). However, other studies found no evidence for changes in initial trust during adolescence (Fett et al., 2014 [ages 13–18]; Güroğlu et al., 2014 [ages 9–18]; Lemmers-Jansen et al., 2019 [ages 13–19]; Lemmers-Jansen et al., 2017 [ages 16–27]; Sweijen et al., 2023 [ages 11–20]; Van de Groep et al., 2018 [ages 12–18]). No studies reported a decrease in initial trust during adolescence.

In its classic form, the trust game is played with an anonymous other to avoid reputation effects. However, several studies have developed variations of the trust game to investigate how initial trust differs for different targets of trust, including ingroup versus outgroup trust. Güroğlu et al. (2014) led participants to play a series of one-shot trust games with four different interaction partners (reciprocal friends, reciprocal antagonists, neutral, and anonymous peers) that were identified using sociometric mapping (ages 9–18). Participants showed higher trust for friends compared to the other three categories, but this effect did not differ with age. A similar study by Burnett Heyes et al. (2015) did report an age-related increase in the extent to which reciprocated (compared to unreciprocated) friendship ties were taken into account in investment decisions (sample 1 ages 13–17; sample 2 ages 13–16). More recently, studies have started to distinguish between different kinds of outgroup members. Sweijen et al. (2023) included a target who is representative of the community, to investigate the role of trust in the broader community (ages 11–20). In this study, adolescents showed the least trust to unknown peers, more to a community member, and most to friends. In this specific age range, the effect of age was nonsignificant, but more studies of this kind are needed to elucidate the development of trust in different members of society. Up till now, the studies suggest that the distinction between ingroup trust and outgroup trust is reflected in trust behavior already early in adolescence, but more subtle differences may continue to emerge during adolescence.

Which factors may account for the observed increase in initial trust during adolescence? Here we summarize three factors that may affect initial trust: perception and valuation of uncertainty, perspective-taking, and social preferences. First, recent studies using computational modeling elucidated the role of uncertainty in the development of initial trust. In a novel information-sampling design, Ma et al. (2022) gave participants (aged 10–24) the opportunity to sequentially gather information about the reciprocation history of their game partners before making their investment decision. Bayesian computational modeling revealed an increase in information sampling from 10 till 16 years, indicative of an increase in perceived uncertainty of the prior beliefs, which leads to more information seeking. The second study showed that reduced risk aversion was an important developmental factor driving the increase in trust during adolescence (Hula et al., 2021 [ages 14–25]). In the context of the trust game, risk aversion refers to the value of the certain amount that is kept, relative to the value of the uncertain return. This study further reported that males showed lower risk aversion in the trust game than females (Hula et al., 2021). A novel and important finding was that socio-economic deprivation was also associated with more risk-averse play. While increased risk-taking in adolescence is sometimes considered harmful, reduced risk aversion in the context of the trust game was beneficial, as it led to higher total payoffs. This observation contributes to a more nuanced view on adolescent risk-taking, by showing that it can also take a positive form such as facilitating the creation of social opportunities (see also Duell and Steinberg (2019)).

Second, the ability and propensity to take another person’s perspective may shape trust behavior in complex ways. On the one hand, better perspective-taking is associated with more prosocial behavior and better social functioning (Tamnes et al., 2018) and may therefore be associated with higher trust. On the other hand, perspective-taking skills may also be employed to protect self-interest, which may be reflected in a stronger adaptation to (anticipated) untrustworthy returns (Sassenrath et al., 2022). Advanced levels of perspective-taking continue to show increases across adolescence (Hollarek and Lee, 2022) and this development may influence trust behavior. Accordingly, performance on a visual perspective-taking task was associated with trust behavior in a sample of 13–18 year olds (Fett, Shergill, et al., 2014). Better performance was associated with higher initial trust, but also with steeper adjustment to an untrustworthy partner, illustrating the dual ways in which perspective-taking can shape trust behavior. Two studies using self-report measures of perspective-taking, however, did not find associations with trust behavior (Sijtsma et al., 2023 [ages 12–16]; Van de Groep et al., 2018 [ages 12–18]), therefore it can be concluded that associations between perspective-taking and trust are sample- and task-dependent.

Finally, social preferences refer to the attitudes toward material payoffs for the self, relative to material payoffs for the other (Fehr and Fischbacher, 2002). An important type of social preference is inequity aversion, which means that people want to increase the material payoff for someone else if this payoff is below an equitable standard, and want to decrease the payoff for the other if this payoff exceeds the equitable standard. The equity norm is established in childhood (Fehr et al., 2008), but during adolescence, this norm is increasingly pitted against the net gain of the payoff (Meuwese et al., 2015, Sutter et al., 2019). The ongoing development of fairness and efficiency concerns may play a role in the development of trust game behavior during adolescence. In line, Westhoff et al. (2020) showed that the age-related increase in initial trust was partly explained by an age-related decrease in disadvantageous inequity aversion (ages 8–23). In other words, with age adolescents were increasingly less concerned about outcomes in which they are worse off than the other player.

In sum, initial trust in the trust game increases or remains stable during adolescence. Studies reporting an increase in trust have generally suggested a linear trend from early up till late adolescence. Early adolescents already distinguish between different targets of trust, including different ingroup and outgroup members, but more studies are needed to delineate the developmental trends in trust toward different targets. Beliefs about trustworthiness tend to become more uncertain during adolescence, which together with a reduced risk aversion may increase investments, in order to gather more information about the trustworthiness of the other. Perspective-taking and inequity aversion influence the decision to trust and may further drive the developmental trends during adolescence.

### 3.2 Neuroimaging studies

The majority of neuroimaging studies of trust used multi-round trust games in which the processes involved in trust decisions include learning on the basis of the feedback from previous rounds. These studies will be discussed under Section 4.2. Trust decisions elicit activity in brain networks related to reward processing (reward network), perspective-taking (default-mode network (DMN)), cognitive control (central executive network (CEN)) and salience detection, including related affective responses (salience network (SAL)) (Krueger and Meyer-Lindenberg, 2019). One recent study used multiple one-shot games with different targets: friends, community members, and unknown peers (Sweijen et al., 2023 [ages 12–23]). In line with the behavioral study (Sweijen et al., 2023), adolescents showed most trust to friends, less trust to community members, and the least trust to unknown peers. Neural results showed that the differentiation between targets was associated with activity in regions of the four brain networks implicated in trust. Recruitment of the DMN implicated in perspective-taking, specifically of the medial prefrontal cortex (mPFC), was higher for friends and community members than for unknown peers, and this effect was most pronounced during no-trust choices. For no-trust choices, brain activity in the mPFC increased with age. Trust to friends was additionally associated with increased activity in posterior brain areas of the DMN, the precuneus, and bilateral temporal parietal junction (TPJ). Activity in the orbitofrontal cortex (OFC), included in the reward network, was higher for trust decisions involving closer targets and this was associated with higher behavioral trust and with self-reported social reward sensitivity. In contrast, activity within regions of the CEN (bilateral dorsolateral prefrontal cortex, dlPFC) and of the SAL (dorsal anterior cingulate cortex (dACC) and anterior insula), showed higher activation during no-trust decisions for friends, and during trust decisions for unknown peers, suggesting that these unexpected decisions require more cognitive control and are viewed as more salient.

In sum, neural mechanisms of initial trust in different targets point to networks associated with reward (reward-network; OFC) and perspective-taking (DMN; mPFC, TPJ, precuneus) involved in trust in close targets and networks associated with cognitive control (CEN; dlPFC) as well as salience detection (SAL; dACC, anterior insula) involved in trust in strangers.

## 4 The development of trust adaptation

### 4.1 Behavioral studies

Once initial trust is conveyed, the feedback from the other can be used to update the prior beliefs, and the adaptation of trust becomes important (see Fig. 1). Several studies have used multiple round games with interaction partners with varying levels of trustworthiness to study age-related changes in the adaptation of trust. Overall, these studies point to an increasing ability to adjust trust on the basis of partner responses during adolescence, with most studies reporting linear changes from early to late adolescence (Sijtsma et al., 2023, Van den Bos et al., 2012, Westhoff et al., 2020). Adolescents also become increasingly better at integrating new information on trustee responses with prior expectations. In the study by Lee et al. (2016), participants played multi-round trust games with three hypothetical partners, for whom prior information about morally good, bad, or neutral behavior was presented (ages 12–18). The actual trust game behavior of the three partners was preprogrammed to be identical and could be described as modestly trustworthy. All age groups adjusted their initial trust behavior in line with the a priori information, and put most trust in the good partner, least in the bad partner. With age, the ability to overcome the prior information and adapt trust behavior improved, as shown by a decrease in trust in the good partner, and an increase in trust in the bad partner.

An as yet unresolved question is whether the development of learning in the trust game differs between trustworthy and untrustworthy interactions. Comparing three age groups (11, 16, and 21 years old), Van den Bos et al. (2012) reported that adaptation to both trustworthy and untrustworthy partners increased with age. Other studies found differential patterns for trustworthy and untrustworthy interactions, but in opposite directions. Westhoff et al. (2020) showed that adaptation to trustworthy interactions increased rapidly in early adolescence, whereas adaptation to untrustworthy interactions changed only slightly (ages 8–23). In contrast, in a longitudinal study, Sijtsma et al. (2023) reported that the adaptation to untrustworthy interactions improved between ages 12 and 16, whereas adaptation to trustworthy partners remained stable. Some studies found gender-specific trajectories, particularly a stronger adaptation to untrustworthy interactions in younger compared to older males (Lemmers-Jansen et al., 2019 [ages 13–19]) and a stronger adaptation to untrustworthy interactions in males compared to females independent of age (Lemmers-Jansen et al., 2017 [ages 16–27]). Presumably, differences in the way the trustworthy and untrustworthy algorithms are programmed are an important factor explaining inconsistencies between studies. In addition, adaptation to trustworthy and untrustworthy interactions may also be asymmetrical in adults. In the study by Li et al. (2021), the (pre-programmed) interaction partners changed their trustworthiness behavior during the game, one from untrustworthy to neutral and the other from trustworthy to neutral interactions. Both adolescents (ages 14–18) and young adults (ages 19–29) showed less adaptation in the negative direction in response to the trustworthy-to-neutral partner and more adaptation in the positive direction in response to the untrustworthy-to-neutral partner. This suggests an asymmetry in which a positive reputation tends to last, despite lower than expected returns, but a negative reputation can be repaired by higher than expected returns (see Bellucci and Park (2020) for further evidence for this asymmetry).

Which factors may contribute to the development of trust adaptation during adolescence? The factors that affect initial trust described above (i.e., perception and valuation of uncertainty, perspective-taking, and social preferences), will continue to affect trust decisions in multi-round games. In addition, an important factor driving the development of trust adaptation may be the process of learning from the signals of the other person. Indeed, two studies using computational modeling found evidence for a development of learning rate. In the study by Westhoff et al. (2020) (ages 8–23), young adolescents continued to update their trust behavior throughout the game whereas older adolescents showed high learning rates in the early rounds. The second study included adolescents (ages 14–18) and young adults (ages 19–29) and this study also reported higher learning rates in the older compared to the younger group (Li et al., 2021).

Another factor that may play a role in the development of trust adaptation is emotion regulation. Lower than expected returns may elicit anger and irritation and lead to a lowering in investments that eventually is costly. Overcoming these negative emotions in order to restore trust may be an important factor underlying mutually cooperative interactions (King-Casas et al., 2008). Emotion regulation continues to develop in adolescence (Silvers, 2022) and an increased ability to regulate negative emotions to untrustworthy responses may contribute to more successful interactions. Indeed, in the study by Van den Bos et al., (2012) the youngest group (age 11) showed a higher tendency to punish untrustworthy returns than the older groups (16 and 21 years), and this was mediated by self-reported feelings of anger. Using computational modeling, irritability in response to low returns was identified as an important factor that negatively impacted on strategic adaptation in the trust game (Hula et al., 2021 [ages 14–25]; Reiter et al., 2023 [ages 14–25]). Of note, higher levels of irritability were found in adolescents who reported adverse family experiences, and this factor explained their attenuated developmental gain in learning to trust in a multi-round trust game (Reiter et al., 2023). This points to the long-lasting effects of adverse childhood experience which may become manifest in a reduced ability to build and maintain mutually trusting relationships.

In sum, multi-round trust games with trustworthy and untrustworthy partners have shown that learning whom to trust and whom not to trust improves during adolescence. Specifically, studies have found evidence for a higher learning rate and an improved ability to overcome initial expectations on the basis of new information in older compared to younger adolescents. However, the literature is inconclusive as to what extent the developmental trajectories of adaptation differs between trustworthy and untrustworthy interactions. Furthermore, the development of more mature emotion regulation skills during adolescence may facilitate strategic adaptation to lower-than-expected returns in middle- and late adolescents instead of an anger-driven tit-for-tat approach in early adolescents.

### 4.2 Neuroimaging studies

Using multi-round trust games, several studies have pointed to a key role for reward-based learning and associated regions of the reward network in trust adaptation. In a study with adolescents and adults, activity in this network (particularly the OFC and the bilateral dorsal striatum (DS)) decreased with age in interactions with a trustworthy partner but not during untrustworthy interactions (Fett, Gromann, et al., 2014 [ages 13–49]). In a recent longitudinal study probing interactions with an untrustworthy partner in early to mid-adolescents, the ventral and dorsal striatal regions of the reward network (ventral striatum (VS) and DS) were activated during investment decisions, independent of age, while activation in the DS during the feedback phase increased with age (Schreuders et al., 2023 [ages 12–16]). At the behavioral level, adolescents became better with increasing age at adapting to the untrustworthy interactions by strategically lowering their investments (Schreuders et al., 2023 [ages 12–16]). Two cross-sectional studies also reported age-related changes in activity in the reward network (DS) during multi-round trust games (Lemmers-Jansen et al., 2019 [ages 13–19]; Lemmers-Jansen et al., 2017 [16–27]). The first study (Lemmers-Jansen et al., 2019) showed an age-related increase in DS activity during interactions with a trustworthy partner in males but an age-related decrease in female participants. The latter study (Lemmers-Jansen et al., 2017) revealed age-related increases in DS activity during the feedback phase in interactions with a trustworthy and with an untrustworthy partner. These findings suggest developmental changes in the recruitment of the reward network during learning to trust, although both age-related increases as well as decreases have been observed. This may tentatively indicate age-related differences in involvement of this network in signaling outcomes and possibly the updating of expectations and prior beliefs.

Moreover, studies in adolescents have reported activation in the DMN associated with perspective-taking during trust decisions. Specifically, age-related increases in the right TPJ during trustworthy interactions were found in two studies (Lemmers-Jansen et al., 2019 [ages 13–19]; Lemmers-Jansen et al., 2017 [ages 16–27]), but in the younger sample in males only. Spanning a smaller age range (ages 12–16), Schreuders et al. (2023) reported activation in the mPFC during untrustworthy interactions, but found no age-related changes.

Neuroimaging studies have further demonstrated the role of cognitive control as indicated by the involvement of the CEN during trust decisions (Lemmers-Jansen et al., 2017; Sijtsma, Lee, van Kesteren, et al., 2023). One study reported age-related increases in the dlPFC, a core region of the CEN, during trustworthy interactions (Lemmers-Jansen et al., 2017 [ages 16–27]). Additionally, in a study in which prior beliefs were manipulated, trustworthy returns by a partner with an untrustworthy reputation elicited activation of the dlPFC (Sijtsma et al., 2023; mean age 17 years), suggesting that cognitive control processes may be specifically recruited in contexts with high uncertainty, such as unexpected feedback.

Finally, untrustworthy interactions may be regarded as more salient and may elicit both aversive reactions, as shown in behavioral studies (Hula et al., 2021, Reiter et al., 2023, Van den Bos et al., 2012), as well as increased conflict monitoring mediated by the SAL. A cross-sectional study reported age-related increases within the dACC, a core region of the SAL, during untrustworthy interactions (Lemmers-Jansen et al., 2019 [ages 13–19]), while a longitudinal study in adolescents between the ages of 12 and 16 years also showed dACC activation during untrustworthy interactions, however no increase with age was observed (Schreuders et al., 2023). Additionally, the study by Schreuders et al. (2023) showed activation in the SAL, specifically within the anterior insula, during feedback from an untrustworthy partner, possibly associated with aversive feelings to negative returns. However, since no measures of mood were included in that study, further studies are required to probe the relevance of anterior insular activity to possible aversive feelings during untrustworthy interactions.

In sum, studies investigating the neural correlates of trust game behavior in adolescent samples have demonstrated the involvement of the reward-network associated with reward-based learning (i.e., VS, DS, OFC) and the CEN implicated in cognitive control (i.e., dlPFC) in the decision and feedback phases of the multi-round trust game. In the decision phase, studies have additionally pointed to activation in the DMN associated with perspective-taking (i.e., TPJ, mPFC). Moreover, untrustworthy interactions elicit activity within the SAL (anterior insula, dACC) related to salience detection, conflict monitoring, and aversion responses. The literature is limited however, and the findings are inconsistent regarding developmental trends in the activation in these areas.

## 5 Discussion

### 5.1 Trust as a form of social reinforcement learning

**Fig. 2:**
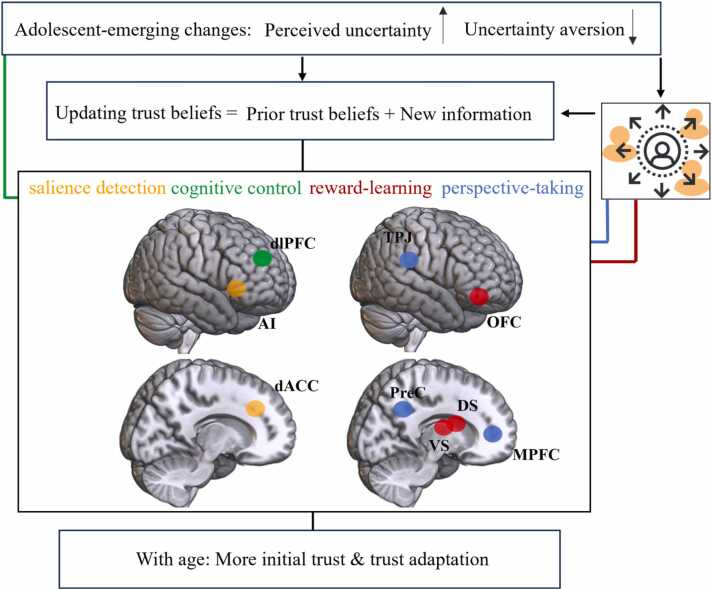
*Model of trust as social reinforcement learning.* Initial trust and adaptation of trust as a form of social reinforcement learning in which trust beliefs are updated by the assimilation of prior trust beliefs with new information. Adolescent-emerging increases of perceived uncertainty and decreases of uncertainty aversion lead to an increased uncertainty of prior beliefs and increased search for new information. The cognitive control network enables flexibly adapting behavior and direct cognitive processing in particular with high perceived uncertainty. Relatedly, the salience network facilitates conflict monitoring and regulates affective responses such as aversion to untrustworthy behavior. The reward-network is involved in signaling differences in outcomes and expectation and consequently updating trust expectations and prior beliefs. Trust learning requires continuous perspective-taking mediated by the default-mode network to infer the intentions of the other facilitating trust beliefs. Social sources are an important source of information, and the social closeness affects the impact of the information on updating of trust beliefs, and related reward-learning and perspective-taking processes. dACC = dorsal anterior cingulate cortex; AI = anterior insula; dlPFC= dorsolateral prefrontal cortex; DS = dorsal striatum; VS = ventral striatum; OFC = orbitofrontal cortex; MPFC = medial prefrontal cortex; PreC = precuneus; TPJ = temporal parietal junction.

The findings on the development of initial trust and adaptation of trust during adolescence align with the general model of social learning in adolescence (see Fig. 2.). An increase in the (perceived) uncertainty of prior trust beliefs together with a reduced risk aversion leads to an increased tendency to probe the trustworthiness of the other through an increase in initial trust. This development is further strengthened by an increased preference for efficiency above equity. The increase in trust is adaptive in the light of the developmental tasks during adolescence, exploring the social world, engaging in new relationships, and achieving independence from caregivers. The development of strategic adaptation to the behavior of the other is facilitated by higher learning rates and better emotion regulation in older compared to younger adolescents. At the neural level, learning to trust is supported by the reward network (ventral and dorsal striatum, orbitofrontal cortex), involved in the signaling of outcomes that are different than expected and the subsequent updating of the expectations and prior beliefs (Schreuders et al., 2023). Strategic adaptation of trust requires cognitive control, in order to monitor and adapt behavior in line with internal goals. This is conceptually similar to model-based reinforcement learning, in which an internal model is used to proactively calculate the value of future actions. The goal-directed nature of trust is supported by neuroimaging studies demonstrating the involvement of the central executive network associated with cognitive control during trust decisions (Fett et al., 2014, Lemmers-Jansen et al., 2019, Lemmers-Jansen et al., 2017). Regions of the central executive network as well as the salience network may be specifically recruited for trust decisions with high perceived uncertainty or which deviate from social norms (Sweijen et al., 2023). Within the framework of model-based reinforcement learning, perspective-taking skills are needed to infer the hidden intentions behind the observable actions of the other person and to continuously update these inferences based on new information. A low return, for example, may be interpreted as a signal that the other is acting out of self-interest, but it may also be interpreted as a warning signal that investments should be increased to establish trust, and the actual intentions may only become clear over time. Several studies reported evidence for a role of perspective-taking in trust, but not all studies confirmed this, showing that the context of the task and the extent towards which perspective-taking is required likely plays an important role. At the neural level, the default-mode network (mPFC, TPJ, precuneus), implicated in perspective-taking, is activated during initial trust and adaptation of trust, and may specifically be recruited during interactions with close or personally-relevant targets. An interesting direction for future research is to further examine how perspective-taking interacts with personal norms and social preferences. This may shed further light on the way perspective-taking may impact trust decisions. Possibly, higher perspective-taking increases trust in individuals who value outcomes for others equal to outcomes for self, and decreases trust in individuals who are concerned with outcomes for self only (see Derks et al. (2015) for some initial evidence). This may be especially relevant since these preferences are mostly likely learned with experiences and can be dependent on social structures, such as family relations and social-economic background (Sutter et al., 2019).

### 5.2 Strengths and limitations of the trust game

The trust game has many strengths. Here we highlight two important features. First, despite its stylized nature, behavior in the trust game reflects behavior in actual social interactions and predicts social functioning. Accordingly, examination of individual differences in trust behavior can be used to better understand typical and atypical adolescent (social) development. In this context, research using the trust game has contributed to our understanding how trust behavior relates to aspects important for adolescent life, such as the creation of peer relationships. A recent study that used the trust game showed that trust is positively related to the degree of social connectedness one has within their friendship network (Sijtsma et al., 2023). Furthermore, the results of a longitudinal study showed that more trust behavior is associated with establishing and maintaining higher quality peer relationships three years later (Reiter et al., 2023). The latter association was stronger for adolescents with adverse family experiences during childhood. For them, interpersonal trust could function as a resilience factor, as showing higher trust enabled these adolescents to form peer relationships of high quality after the experience of adverse social interactions with family members (Reiter et al., 2023). Further, the trust game can contribute to a better understanding of social-cognitive functioning in adolescents with atypical development. Research has shown atypical levels of trust behavior and patterns of neural activity in adolescents with depressive symptoms (Mellick et al., 2019), externalizing behavior problems (Mellick et al., 2019, Sharp et al., 2011, Sharp et al., 2011), and psychotic symptoms (Fett et al., 2016, Hanssen et al., 2021, Lemmers-Jansen et al., 2019, Lemmers-Jansen et al., 2020). Overall, these studies point to the relevance of the trust game to use it as a proxy of social decision-making within the context of a simulated social exchange to better understand adolescent social functioning and psychopathology.

Second, the context-free and quantitative nature of the trust game provides the possibility to manipulate factors of interest while at the same time ensuring experimental rigor. As such, the trust game has been adapted to study prior expectations and adaptation to feedback, and the integration of these. A particularly fruitful approach is computational modeling to elucidate the mechanisms underlying trust decisions. Using this approach, each participant is individually characterized by quantitative scores on relevant factors such as prior beliefs, updating of prior beliefs, risk preferences and social preferences which can then be related to relevant individual characteristics and to neural correlates.

However, these strengths co-occur with important limitations. Conceptually, the trust game assesses a specific form of trust, namely trust *in reciprocity*. That is, the investment in the trust game is at least in part driven by the expectation of a back transfer. In daily life however, many instances of trust can occur, in which there is no expectation of direct reciprocity. For example, an adolescent may disclose private information to her friend, trusting her friend not to share this information with others, but without expecting a direct benefit from this behavior. Trust without direct reciprocity is also common in interactions with strangers, for example, when trusting someone to look after your luggage for a short time. It is also important to note that in daily life, trust in strangers is often transactional and governed by regulations, which will impact the motives underlying trust decisions. Furthermore, it would be useful to consider trust behavior beyond monetary exchange. Even though social rather than financial motives drive trust game behavior, key aspects of trust for adolescents, such as keeping promises, keeping secrets, standing up for friends and being honest, are not particularly salient in the trust game. A different conceptual consideration is that behavior in the trust game may be driven by motives unrelated to trust. The trustor, for example, may invest because of efficiency motives (i.e., the desire to maximize the gain in the game regardless of the return by the trustee), or by other-regarding preferences such as advantageous inequity aversion (see also Section 3.1).

Methodologically, behavior in the trust game is sensitive to relatively minor changes in the setup of the game. Examples are the size of the multiplier, which is three in most versions of the trust game, but is sometimes set to a lower or higher number, the use of the strategy method or actual decisions; or framing the trustee as partner or opponent (Alós-Ferrer and Farolfi, 2019). This means that to increase comparability of studies, variations on the standard game should be carefully considered and only implemented if relevant to the study goals.

Finally, for adolescents, trust involves many facets that are not included in the trust game, such as keeping promises, keeping secrets, and standing up for your friends. It would be useful to consider how measures of trust can better reflect these facets. One way would be to still use the format of the trust game, but manipulate the trustworthiness levels of different targets of trust by letting them display these forms of behavior. Another way would be to introduce another form of exchange between the trustor and the target of trust, such as personal or otherwise precious information that can be shared or kept. However, this would pose specific challenges, for example, how to make a sharing decision beneficial to both parties and how to quantify the level of trust and trustworthiness. Lastly, behavior in the trust game could be usefully related to trust behavior in daily life, such as assessed with Ecological Momentary Assessment or diary methods (Weiss et al., 2022).

## 6 Conclusions and Future research

The ability to build trusting relationships with strangers opens up opportunities beyond one's immediate network. The finding that coming from a disadvantaged background can have a negative impact on the development of this skill makes it even more important to tailor the study of trust to understand this diversity. Initial research highlights how social background characteristics may influence the development of trust. Coming from a disadvantaged social-economic background may impede the developmental trajectory through the effects on risk aversion (Hula et al., 2021), and a history of adverse family experiences may do so through decreased emotion regulation (Pitula et al., 2017, Reiter et al., 2023). This underscores the importance of including and measuring different forms of diversity in developmental studies of trust. Being able to trust provides important opportunities for adolescents and the negative effects of adverse environments may trigger vicious circles of mutual distrust. In this context, an important avenue for future research is to study trust in representatives of different societal institutions. This builds on the work of Sweijen et al. (2023) who introduced a member of a community group as a target of trust. Combined with a precise mapping of the experience of psychological and social adversity, this type of research could further elucidate the mechanisms underlying associations between negative prior experiences and the building of trust, and possibly also how ruptured trust can be repaired.

Future research should target connectivity and interplay between the reward network, default-mode network, central executive network, and salience network that may underlie trust interactions. Intrinsic connectivity between and within brain networks shows developmental changes across adolescence (Grayson and Fair, 2017, Gu et al., 2015, Marek et al., 2015, Stevens, 2016, Váša et al., 2020) and has been related to the integration and specialization of brain networks which may facilitate maturation of cognitive processes (Luna et al., 2015, Marek et al., 2015). In young adults, intrinsic connectivity within the default-mode network was associated with inter-individual differences in the propensity to trust, while between network interactions of the default-mode network, salience network, and central executive network were associated with the propensity to reciprocate (Belluci et al., 2019). Additionally, while studies on connectivity during trust interactions are scarce, one study showed increased task-based connectivity of the default-mode network with a prefrontal region and parietal cortex during reciprocated compared to violated trust from friends relative to strangers in young adults (Fareri et al., 2020). These limited studies in young adults indicate the relevance of inter-brain network connectivity in trust. Targeting functional connectivity between the reward network, default-mode network, central executive network, and salience network in relation to trust may shed light on how network interactions underlie the complex interplay between reward processes, perspective-taking, cognitive control, and affective responses during trust interactions in adolescents. In this further investigation, the role of the anterior insula in the development of trust across adolescence should be elucidated, as this region is thought to serve as a key node in coordinating the dynamic switching between the networks underlying trust (Krueger and Meyer-Lindenberg, 2019). Moreover, next to developmental changes in functional connectivity, development of structural connectivity may also relate to changes in trust behavior. There is emerging evidence supporting the association between white matter pathways within networks supporting social processing, including the DMN, and social cognition (Wang et al., 2018). Future studies should target the association between structural connectivity and trust across adolescence utilizing Diffusion Tensor Imaging in addition to functional MRI.

This review further highlights the need to investigate developmental trends in trust behavior across adolescence. The limited evidence so far mostly points to linear changes in initial trust and trust adaptation, and their underlying neural mechanisms, from early adolescence well into early adulthood. However, specific mechanisms associated with trust behavior, such as risk aversion and emotion regulation, may well show nonlinear trends. For example, the development of risk taking shows an inverted U-shaped trajectory, with peeks in middle or late adolescence dependent on the type of risk and whether risk-taking propensity or real world risk taking is assessed (Duell et al., 2018). An important question is further which aspects of trust behavior are driven by changes in pubertal hormones and which are age-related. Pubertal development has been linked to changes in brain areas implicated in the processing of social and motivational signals as well as in social and affective behavior (Pfeifer and Allen, 2021). For example, there is evidence for an increase in emotional instability associated with the onset of puberty (Bailen et al., 2019). The finding discussed above that young adolescents show higher anger and irritability in response to untrustworthy returns compared to older adolescents (Van den Bos et al., 2012) accords with this evidence and could be further explored in studies assessing pubertal maturation.

Future research should also use novel ways to examine and interpret trust in contemporary society. Today’s youth grow up in a digital world. Connections can be initiated and maintained using digital platforms without constraints due to geographic distance. This poses specific challenges and opportunities to the building of trust. Digital communication in close relationships may be confounded by blurred or biased representations of reality and by the incomplete nonverbal information that normally supports communication. Digital communications with strangers may be even more risky because of the possibility of catfishing or fraud. A pressing question is therefore how trust and adaptation of trust develop in online environments (Fogel and Nehmad, 2009, Kopton et al., 2013). This may require developing trust games that mimic these online interactions, in terms of the information that is presented about the target of trust, but also about the behavior of other online visitors, since seeking and using information on the behavior of others may be a key determinant of online trust behavior. However, digital communication also provides many opportunities. There are many societal challenges facing youth that are global and require collaborations beyond borders, such as climate change policies, social inequalities, and the recent COVID-19 pandemic. Overcoming these challenges requires collaboration at multiple levels and the young generation may be the glue for society that is needed to open up novel possibilities for ways out of the multiple societal crisis based on trust and reciprocity (Falk and Bassett, 2017).

## CRediT authorship contribution statement

**Lydia Krabbendam:** Writing – original draft, Funding acquisition, Conceptualization. **Eveline A. Crone:** Writing – review & editing, Funding acquisition, Conceptualization. **Hester Sijtsma:** Writing – review & editing, Visualization, Conceptualization. **Mariët Van Buuren:** Writing – review & editing, Visualization, Conceptualization.

## Declaration of Competing Interest

The authors declare that they have no known competing financial interests or personal relationships that could have appeared to influence the work reported in this paper.

## References

[bib1] Alós-Ferrer C., Farolfi F. (2019). Trust games and beyond. Front. Neurosci..

[bib2] Bailen N.H., Green L.M., Thompson R.J. (2019). Understanding emotion in adolescents: a review of emotional frequency, intensity, instability, and clarity. Emot. Rev..

[bib3] Bellucci G., Park S.Q. (2020). Honesty biases trustworthiness impressions. J. Exp. Psychol.: Gen..

[bib4] Belluci Hahn, Deshpande Krueger (2019). Functional connectivity of specific resting-state networks predicts trust and reciprocity in the trust game. Cogn., Affect., Behav. Neurosci..

[bib5] Berg J., Dickhaut J., McCabe K. (1995). Trust, reciprocity, and social history. Games Econ. Behav..

[bib6] Bowlby, J.Attachment. Attachment and loss: Vol. 1. Loss. New York: Basic Books..

[bib7] Burnett Heyes S., Jih Y.-R., Block P., Hiu C.-F., Holmes E.A., Lau J.Y.F. (2015). Relationship reciprocation modulates resource allocation in adolescent social networks: developmental effects. Child Dev..

[bib8] De Neys W., Hopfensitz A., Bonnefon J.-F. (2015). Adolescents gradually improve at detecting trustworthiness from the facial features of unknown adults. J. Econ. Psychol..

[bib9] Delgado E., Serna C., Martínez I., Cruise E. (2022). Parental attachment and peer relationships in adolescence: a systematic review. Int. J. Environ. Res. Public Health.

[bib10] Delhey J., Welzel C. (2012). Generalizing trust: how outgroup-trust grows beyond ingroup-trust. World Values Res..

[bib11] Derks J., Van Scheppingen M.A., Lee N.C., Krabbendam L. (2015). Trust and mindreading in adolescents: the moderating role of social value orientation. Front. Psychol..

[bib12] Duell N., Steinberg L., Icenogle G., Chein J., Chaudhary N., Di Giunta L., Chang L. (2018). Age patterns in risk taking across the world. Journal of Youth and Adolescence.

[bib13] Duell N., Steinberg L. (2019). Positive risk taking in adolescence. Child Dev. Perspect..

[bib14] Erikson, E.H. (1963). Childhood and society (2 ed.). WW Norton & Company.

[bib15] Fairley K., Sanfey A., Vyrastekova J., Weitzel U. (2016). Trust and risk revisited. J. Econ. Psychol..

[bib16] Falk E.B., Bassett D.S. (2017). Brain and social networks: fundamental building blocks of human experience. Trends Cogn. Sci..

[bib17] Fareri D.S., Smith D.V., Delgado M.R. (2020). The influence of relationship closeness on default-mode network connectivity during social interactions. Soc. Cogn. Affect. Neurosci..

[bib18] Feeney B.C., Cassidy J., Ramos-Marcuse F. (2008). The generalization of attachment representations to new social situations: predicting behavior during initial interactions with strangers. J. Personal. Soc. Psychol..

[bib19] Fehr E., Bernhard H., Rockenbach B. (2008). Egalitarianism in young children. Nature.

[bib20] Fehr E., Fischbacher U., Kosfeld M. (2005). Neuroeconomic foundations of trust and social preferences: initial evidence. Am. Econ. Rev..

[bib21] Fehr E., Fischbacher U. (2002). Why social preferences matter –the impact of non-selfish motives on competition, cooperation and incentives. Econ. J..

[bib22] Fett A.-K.J., Gromann P.M., Giampietro V., Shergill S.S., Krabbendam L. (2014). Default distrust? An fMRI investigation of the neural development of trust and cooperation. Soc. Cogn. Affect. Neurosci..

[bib23] Fett A.-K.J., Shergill S.S., Gromann P.M., Dumontheil I., Blakemore S.-J., Yakub F., Krabbendam L. (2014). Trust and social reciprocity in adolescence – a matter of perspective-taking. J. Adolesc..

[bib24] Fett A.-K.J., Shergill S., Korver-Nieberg N., Yakub F., Gromann P., Krabbendam L. (2016). Learning to trust: trust and attachment in early psychosis. Psychol. Med..

[bib25] Flanagan C.A., Stout M. (2010). Developmental patterns of social trust between early and late adolescence: age and school climate effects. J. Res. Adolesc..

[bib26] Fogel J., Nehmad E. (2009). Internet social network communities: risk taking, trust, and privacy concerns. Comput. Hum. Behav..

[bib27] Glaeser E.L., Laibson D.I., Scheinkman J.A., Soutter C.L. (2000). Measuring trust. Q. J. Econ..

[bib28] Grayson D.S., Fair D.A. (2017). Development of large-scale functional networks from birth to adulthood: A guide to the neuroimaging literature. Neuroimage.

[bib29] Gu S., Satterthwaite T.D., Medaglia J.D., Yang M., Gur R.E., Gur R.C., Bassett D.S. (2015). Emergence of system roles in normative neurodevelopment. Proceedings of the National Academy of Sciences.

[bib30] Güroğlu B., van den Bos W., Crone E.A. (2014). Sharing and giving across adolescence: an experimental study examining the development of prosocial behavior. Front. Psychol..

[bib31] Guyer A.E. (2020). Adolescent psychopathology: The role of brain-based diatheses, sensitivities, and susceptibilities. Child Dev. Perspect..

[bib32] Hancock P., Kessler T.T., Kaplan A.D., Stowers K., Brill J.C., Billings D.R., Schaefer K.E., Szalma J.L. (2023). How and why humans trust: a meta-analysis and elaborated model. Front. Psychol..

[bib33] Hanssen E., Van Buuren M., Van Atteveldt N., Lemmers-Jansen I.L.J., Fett A.-K.J. (2021). Neural, behavioural and real-life correlates of social context sensitivity and social reward learning during interpersonal interactions in the schizophrenia spectrum. Aust. N. Z. J. Psychiatry.

[bib34] Harbaugh, W.T., Krause, K., & Liday Jr., S.G. (2003). Bargaining by children. Working paper. https://scholarsbank.uoregon.edu/xmlui/handle/1794/86.

[bib35] Hofmans L., Van den Bos W. (2022). Social learning across adolescence: a Bayesian neurocognitive perspective. Dev. Cogn. Neurosci..

[bib36] Hollarek M., Lee N.C. (2022). Current understanding of developmental changes in adolescent perspective taking. Curr. Opin. Psychol..

[bib37] Hula A., Moutoussis M., Will G.-J., Kokorikou D., Reiter A.M., Ziegler G., Consortium N., Bullmore E., Jones P.B., Goodyer I., Fonagy P., Montague P.R., Dolan R.J. (2021). Multi-round trust game quantifies inter-individual differences in social exchange from adolescence to adulthood. Comput. Psychiatry.

[bib38] Integrated Values Surveys (2022). https://ourworldindata.org/grapher/interpersonal-trust-vs-income-inequality.

[bib39] Joiner J., Piva M., Turrin C., Chang S.W.C. (2017). Social learning through prediction error in the brain. NPJ Sci. Learn..

[bib40] Jordahl H., Svendsen G., Svendsen G. (2007). Handbook of Social Capital..

[bib41] King-Casas B., Sharp C., Lomax-Bream L., Lohrenz T., Fonagy P., Montague P.R. (2008). The rupture and repair of cooperation in borderline personality disorder. Science.

[bib42] Kochanska G., Boldt L.J., Goffin K.C. (2019). Early relational experience: A foundation for the unfolding dynamics of parent-child socialization. Child Dev. Perspect..

[bib43] Kopton I., Sommer J., Winkelmann A., Riedl R., Kenning P. (2013). Users' trust building processes during their initial connecting behavior in social networks: behavioral and neural evidence. Proc. Int. Conf. Inf. Syst..

[bib44] Krueger F., Meyer-Lindenberg A. (2019). Toward a model of interpersonal trust drawn from neuroscience, psychology, and economics. Trends Neurosci..

[bib45] Lee N.C., Jolles J., Krabbendam L. (2016). Social information influences trust behaviour in adolescents. J. Adolesc..

[bib46] Lemmers-Jansen I.L.J., Fett A.-K.J., Hanssen E., Veltman D.J., Krabbendam L. (2019). Learning to trust: social feedback normalizes trust behavior in first-episode psychosis and clinical high risk. Psychol. Med..

[bib47] Lemmers-Jansen I.L.J., Fett A.-K.J., Shergill S.S., Van Kesteren M.T.R., Krabbendam L. (2019). Girls-boys: An investigation of gender differences in the behavioral and neural mechanisms of trust and reciprocity in adolescence. Front. Hum. Neurosci..

[bib48] Lemmers-Jansen I.L.J., Fett A.-K.J., van Os J., Veltman D.J., Krabbendam L. (2020). Trust and the city: Linking urban upbringing to neural mechanisms of trust in psychosis. Aust. N. Z. J. Psychiatry.

[bib49] Lemmers-Jansen I.L.J., Krabbendam L., Veltman D.J., Fett A.-K.J. (2017). Boys vs. girls: Gender differences in the neural development of trust and reciprocity depend on social context. Dev. Cogn. Neurosci..

[bib50] Li S., Hao X., Mei Y., Cheng Y., Sun N., Qu C. (2021). How adolescents and adults learn about changes in the trustworthiness of others through dynamic interaction. Front. Psychol..

[bib51] Luna B., Marek S., Larsen B., Tervo-Clemmens B., Chahal R. (2015). An integrative model of the maturation of cognitive control. Annual review of neuroscience.

[bib52] Ma I., Westhoff B., van Duijvenvoorde A. (2022). Uncertainty about others’ trustworthiness increases during adolescence and guides social information sampling. Sci. Rep..

[bib53] Marek S., Hwang K., Foran W., Hallquist M.N., Luna B. (2015). The contribution of network organization and integration to the development of cognitive control. PLoS Biol.

[bib54] Mayer R.C., Davis J.H., Schoorman F.D. (1995). An integrative model of organizational trust. Acad. Manag. Rev..

[bib55] Mellick W., Sharp C., Ernst M. (2019). Depressive adolescent girls exhibit atypical social decision-making in an iterative trust game. J. Soc. Clin. Psychol..

[bib56] Mellick W., Sharp C., Sumlin E. (2019). Trust and general risk-taking in externalizing adolescent inpatients versus non-externalizing psychiatric controls. Scand. J. Child Adolesc. Psychiatry Psychol..

[bib57] Meuwese R., Crone E.A., De Rooij M., Güroğlu B. (2015). Development of equity preferences in boys and girls across adolescence. Child Dev..

[bib58] Misztal B.A. (1996).

[bib59] Nelson E.E., Jarcho J.M., Guyer A.E. (2016). Social re-orientation and brain development: an expanded and updated view. Dev. Cogn. Neurosci..

[bib60] OECD (2015).

[bib61] Pfeifer J.H., Allen N.B. (2021 Jan 15). Puberty initiates cascading relationships between neurodevelopmental, social, and internalizing processes across adolescence. Biol. Psychiatry.

[bib62] Pitula C.E., Wenner J.A., Gunnar M.R., Thomas K.M. (2017). To trust or not to trust: social decision-making in post-institutionalized, internationally adopted youth. Dev. Sci..

[bib63] Poulin M.J., Haase C.M. (2015). Growing to trust: evidence that trust increases and sustains well-being across the life span. Soc. Psychol. Personal. Sci..

[bib64] Putnam R.D. (2000). Bowling alone: the collapse and revival of American community. Simon Schuster.

[bib65] Reiter A.M., Hula A., Vanes L., Hauser T.U., Kokorikou D., Goodyer I.M., Consortium N., Fonagy P., Moutoussis M., Dolan R.J. (2023). Self-reported childhood family adversity is linked to an attenuated gain of trust during adolescence. Nat. Commun..

[bib66] Sassenrath C., Vorauer J.D., Hodges S.D. (2022). The link between perspective-taking and prosociality — not as universal as you might think. Curr. Opin. Psychol..

[bib67] Schreuders E., van Buuren M., Walsh R., Sijtsma H., Hollarek M., Lee N.C., Krabbendam L. (2023). Learning whom not to trust across early and middle adolescence: a longitudinal neuroimaging study to trusting behavior involving an uncooperative other. Child Dev..

[bib68] Sharp C., Burton P.C., Ha C. (2011). Better the devil you know”: a preliminary study of the differential modulating effects of reputation on reward processing for boys with and without externalizing behavior problems. Eur. Child Adolesc. Psychiatry.

[bib69] Sharp C., Carolyn H., Fonagy P. (2011). Get them before they get you: trust, trustworthiness, and social cognition in boys with and without externalizing behavior problems. Dev. Psychopathol..

[bib70] Sijtsma H., Lee N., Braams B., Hollarek M., Walsh R., van Buuren M., Krabbendam L. (2023). The development of adolescent trust behavior. J. Exp. Child Psychol..

[bib71] Sijtsma H., Lee N., van Kesteren M., Braams B., van Atteveldt N., Krabbendam L., van Buuren M. (2023). The effect of incorrect prior information on trust behavior in adolescents. Neuropsychologia.

[bib72] Sijtsma H., van Buuren M., Hollarek M., Walsh R.J., Lee N.C., Braams B.R., Krabbendam L. (2023). Social network position, trust behavior, and neural activity in young adolescents. Neuroimage.

[bib73] Silvers J.A. (2022). Adolescence as a pivotal period for emotion regulation development. Curr. Opin. Psychol..

[bib74] Simpson J.A. (2007). Psychological foundations of trust. Curr. Dir. Psychol. Sci..

[bib75] Stevens M.C. (2016). The contributions of resting state and task-based functional connectivity studies to our understanding of adolescent brain network maturation. Neuroscience & Biobehavioral Reviews.

[bib76] Stolle D. (2002). Trusting strangers – The concept of generalized trust in perspective. Österreichische Z. F. üR. Polit..

[bib77] Sutter M., Kocher M.G. (2007). Trust and trustworthiness across different age groups. Games Econ. Behav..

[bib78] Sutter M., Zoller C., Glätzle-Rützler D. (2019). Economic behavior of children and adolescents–A first survey of experimental economics results. Eur. Econ. Rev..

[bib79] Sutton T.E. (2019). Review of attachment theory: familial predictors, continuity and change, and intrapersonal and relational outcomes. Marriage Fam. Rev..

[bib80] Sweijen S.W., Te Brinke L.W., van de Groep S., Crone E.A. (2023). Adolescents' trust and reciprocity toward friends, unknown peers, and community members. J. Res. Adolesc..

[bib81] Sweijen S.W., Van de Groep S., Te Brinke L.W., Fuligni A.J., Crone E.A. (2023). Neural mechanisms underlying trust to friends, community members, and unknown peers in adolescence. J. Cogn. Neurosci..

[bib82] Tamnes C.K., Overbye K., Ferschmann L., Fjell A.M., Walhovd K.B., Blakemore S.-J., Dumontheil I. (2018). Social perspective taking is associated with self-reported prosocial behavior and regional cortical thickness across adolescence. Dev. Psychol..

[bib83] Van de Groep S., Meuwese R., Zanolie K., Güroğlu B., Crone E.A. (2018). Developmental changes and individual differences in trust and reciprocity in adolescence. J. Res. Adolesc..

[bib84] Van den Bos W., Van Dijk E., Crone E.A. (2012). Learning whom to trust in repeated social interactions: a developmental perspective. Group Process. Inter. Relat..

[bib85] Van den Bos W., Westenberg M., Van Dijk E., Crone E.A. (2010). Development of trust and reciprocity in adolescence. Cogn. Dev..

[bib86] Váša F., Romero-Garcia R., Kitzbichler M.G., Seidlitz J., Whitaker K.J., Vaghi M.M., Bullmore E.T. (2020). Conservative and disruptive modes of adolescent change in human brain functional connectivity. Proceedings of the National Academy of Sciences.

[bib87] Wang Y., Metoki A., Alm K.H., Olson I.R. (2018). White matter pathways and social cognition. Neurosci. Biobehav. Rev..

[bib88] Weiss A., Burgmer P., Hofmann W. (2022). The experience of trust in everyday life. Curr. Opin. Psychol..

[bib89] Westhoff B., Molleman L., Viding E., Van den Bos W., Van Duijvenvoorde A.C.K. (2020). Developmental asymmetries in learning to adjust to cooperative and uncooperative environments. Sci. Rep..

